# Draft Genome Sequence of Methicillin-Resistant *Staphylococcus epidermidis* Strain ET-024, Isolated from an Endotracheal Tube Biofilm of a Mechanically Ventilated Patient

**DOI:** 10.1128/genomeA.00527-14

**Published:** 2014-05-29

**Authors:** Ilse Vandecandelaere, Filip Van Nieuwerburgh, Dieter Deforce, Hans J. Nelis, Tom Coenye

**Affiliations:** a Laboratory of Pharmaceutical Microbiology, Ghent University, Ghent, Belgium; b Laboratory of Pharmaceutical Biotechnology, Ghent University, Ghent, Belgium

## Abstract

*Staphylococcus epidermidis* strain ET-024 was isolated from a biofilm on an endotracheal tube of a mechanically ventilated patient. This strain is resistant to methicillin, and the draft genome sequence shares some characteristics with other nosocomial *S. epidermidis* strains (such as *S. epidermidis* RP62A).

## GENOME ANNOUNCEMENT

*Staphylococcus epidermidis* is a commensal skin bacterium which is also recognized as an opportunistic pathogen (1, 2). The majority of infections caused by *S. epidermidis* are associated with the presence of indwelling medical devices, and these infections are related to the ability of *S. epidermidis* to form biofilms (2, 3).

We sequenced the genome of *S. epidermidis* isolate ET-024, recovered from an endotracheal tube biofilm of a mechanically ventilated patient (4). This strain is susceptible to vancomycin (MIC, 2 µg/ml), erythromycin (MIC, 0.25 µg/ml) and tobramycin (MIC, <2 µg/ml) but resistant to methicillin (MIC, 0.5 µg/ml) (5). Sequencing was performed using an Illumina MiSeq and paired reads (7,865,978) of 250 bp were obtained. The high-quality filtered reads were mapped against the *S. epidermidis* RP62A genome using CLC Genomics Workbench 6.5.2 (CLC Bio, Aarhus, Denmark), and in this way the reads were assembled. The RAST server was used to annotate the consensus sequence (6). The draft genome consists of 2,616,532 bp, 110 contigs, 2,161 coding sequences, 85 predicted RNAs, and 374 subsystems. The guanine-cytosine content is 32.3% (7).

Genome sequence analysis demonstrated the presence of the *ica* operon (*icaABCDR*). A major component of the matrix of staphylococcal biofilms is a polymer of β-1,6-linked *N*-acetylglucosamine (PIA), which is formed by the products of 4 genes (*icaABCD*) (8). Also, genes encoding proteins involved in biofilm formation (such as *atlE*, *sasG*, *epb, aap*, and *sdrE*) were identified (2).

Strain ET-024 carries a set of genes encoding proteins involved in stress responses. Proteins which play a role in choline and betaine uptake (glycine betaine is an efficient osmolyte) and betaine biosynthesis (*betA, betB, betT*, and *opuD*) are encoded in the genome of ET-024 (9). Protection against oxidative stress (*sodA* and *sodB*), heat (*dnaJ*, *dnaK*, and *grpE*), and cold shock (*cspA* and *cspC*) (10) is also encoded in the genome of ET-024.

Furthermore, proteins conferring resistance to toxic compounds such as cobalt-zinc-cadmium (*czrB* and *czrD*), mercury (*mir* and *merA*), arsenic (*arsABCDR*), and cadmium (*cadC* and *cadD*) are encoded in the ET-024 genome. Also, this strain carries genes encoding resistance to antibiotics, including teicoplanin (*tcaABR*), fosfomycin (*fosB*), and methicillin (*mecR1*, *mecI*, and *mecA*). In addition, genes encoding multidrug resistance proteins A and B are present in the genome of ET-024. The exact function of these proteins in Gram-positive bacteria is still unknown (11).

The *arcABCD* genes, collectively referred to as the arginine catabolic element (*ACME*) cluster, are also present in the genome of *S. epidermidis* ET-024 (12). Although the precise function of *ACME* in staphylococci has not yet been determined, several studies have shown that *ACME* improves fitness and the ability to colonize mucosae (12). The *opp* gene (*oppABCDF*) cluster is also present (13).

Altogether, the presence of genes involved in biofilm formation and antibiotic resistance in the genome of ET-024 suggests that *S. epidermidis* is more than a harmless commensal bacterium.

### Nucleotide sequence accession numbers.

This whole-genome shotgun project has been deposited at DDBJ/EMBL/GenBank under the accession number JGVL00000000. The version described in this paper is version JGVL01000000.
